# Antibiotics Attenuate Methamphetamine-Induced Hepatotoxicity by Regulating Oxidative Stress and TLR4/MyD88/Traf6 Axis

**DOI:** 10.3389/fphar.2021.716703

**Published:** 2021-07-26

**Authors:** Li-Jian Chen, Jie-Tao He, Ming Pan, Jia-Li Liu, Kai-Kai Zhang, Jia-Hao Li, Li-Bin Wang, Ling-Ling Xu, Yu-Kui Chen, Qin-Yao Zhang, Dong-Ri Li, Jing-Tao Xu, Xiao-Li Xie

**Affiliations:** ^1^ Department of Forensic Pathology, School of Forensic Medicine, Southern Medical University, Guangzhou, China; ^2^ Department of Basic Medicine and Biomedical Engineering, School of Medicine, Foshan University, Foshan, China; ^3^ Department of Anesthesiology, Dalian Municipal Central Hospital, Dalian, China; ^4^ Department of Toxicology, School of Public Health (Guangdong Provincial Key Laboratory of Tropical Disease Research), Southern Medical University, Guangzhou, China; ^5^ Department of Forensic Evidence Science, School of Forensic Medicine, Southern Medical University, Guangzhou, China; ^6^ Department of Forensic Clinical Medicine, School of Forensic Medicine, Southern Medical University, Guangzhou, China

**Keywords:** methamphetamine, antibiotics, hepatotoxicity, RNA-seq, RT-qPCR

## Abstract

Methamphetamine (METH) is a major psychostimulant drug of abuse worldwide, and its neurotoxicity has been studied extensively. In addition to neurotoxicity, METH can also induce hepatotoxicity. The underlying mechanism of intestinal microorganisms in METH-induced hepatotoxicity remains unclear. In this study, mice have received antibiotics intragastrically or PBS once each day for 1 week, followed by METH or saline. The antibiotics attenuated METH-induced hepatotoxicity as evidenced by histopathological observation and biochemical analysis; furthermore, they alleviated METH-induced oxidative stress. The effect of antibiotics on METH-induced hepatotoxicity was investigated using RNA-sequencing (RNA-seq). The RNA-seq results demonstrated that antibiotics could regulate 580 differentially expressed genes (DEGs), of which 319 were upregulated after METH treatment and then downregulated with antibiotic pretreatment and 237 were first downregulated after METH administration and then upregulated after antibiotic pretreatment, in addition to 11 upregulated and 13 downregulated ones simultaneously in METH and antibiotic-pretreated groups. RNA-seq analyses revealed that TLR4 is one of the hub genes. Western blot analysis indicated that antibiotics inhibited the increase of TLR4, MyD88 and Traf6 induced by METH. This research suggests that antibiotics may play an important role in preventing METH-induced liver injury by regulating oxidative stress and TLR4/MyD88/Traf6 axis, though further investigation is required.

## Highlights

Methamphetamine (METH) induces hepatotoxicity in mice.
Antibiotics alleviate METH-induced hepatotoxicity by regulating oxidative stress and TLR4/MyD88/Traf6 pathway.
Clearance of microbiota by antibiotics suggested that gut flora may involve in protecting antibiotic preconditioning on METH-mediated hepatotoxicity.

## Introduction

Abuse of METH has become a major worldwide health problem (Xu et al., 2019; Xu and Liu, 2019). As known, METH is harmful to multiple organs (e.g., the brain, heart, liver, lung, kidney, and spleen), and the current research mainly focuses on its neurotoxicity (Liu et al., 2017; Moratalla et al., 2017; Zhang et al., 2017; Yang et al., 2018), and METH-induced hepatic injury has recently been studied (Dias Da Silva et al., 2013; Halpin et al., 2013). Previous studies found that globulin increased, albumin decreased, and albumin/globulin decreased in METH-abuser serum, indicating that METH would induce hepatic disease and inflammation (Zhao et al., 2020). Eskandari et al. reported that METH cytotoxicity was related to oxidative stress and subsequent mitochondrial membrane conformation changes and cytochrome c release into the cytosol (Eskandari et al., 2014). However, preconditioning of chlorogenic and caftaric acids could prevent liver toxicity and oxidative stress induced by METH injections (Koriem and Soliman, 2014). Similarly, our team demonstrated that METH induced hepatotoxicity by inducing cell cycle arrest and activating apoptosis (Wang et al., 2017) and luteolin exerted protective effects against METH hepatotoxicity by suppressing apoptosis, autophagy, and inflammation in rats (Qu et al., 2020; Zhang et al., 2021).

Accumulating evidence suggested that intestinal flora overgrowth might improve translocation of enteric bacteria and their metabolites to the liver through the portal venous system and lead to inflammation, oxidative stress, and other liver diseases (Berg and Garlington, 1979; Albillos, 2003; Meng et al., 2018). Intestinal microbiological disorders are crucial for developing liver diseases, such as non-alcoholic fatty liver disease (NAFLD), alcoholic liver disease (ALD), cirrhosis, non-alcoholic steatohepatitis (NASH), and viral hepatitis (Milosevic et al., 2019; Albhaisi et al., 2020). Our previous studies have demonstrated that METH induces a decrease in the abundance of intestinal probiotics in the gut and increases the conditionally pathogenic bacteria with pro-inflammatory effects (Chen et al., 2021). Using antibiotics in mice liver disease models clearly demonstrates that targeting intestinal flora can alleviate hepatic inflammation by reducing lipopolysaccharide transport to the liver (Tripathi et al., 2018; Mu and Zhu, 2019). Oral non-absorbable antibiotics can reduce inflammation caused by bacterial translocation (Bajaj et al., 2018; Mendoza et al., 2020). Inflammation was reported to play a significant function in the pathophysiology of neuropsychiatric diseases, and minocycline has potent neuroprotective and anti-inflammatory effects that reduce behavior and dopaminergic neurotoxicity in mice after METH treatment (Hashimoto, 2008; Hashimoto et al., 2013). However, the exact mechanisms of antibiotics on METH-induced hepatotoxicity have not been elucidated.

Herein, mice were pretreated with non-absorbable antibiotics, followed by METH injection. Histopathology and biochemical analyses were conducted to determine hepatic damage, and RNA-seq was performed for a potential mechanism of the protective effects of antibiotic pretreatment in METH. Overall, our findings may provide novel evidence for prevention and therapy of METH-induced hepatotoxicity.

## Materials and Methods

### Chemicals

METH was purchased from the National Institute for the Control of Pharmaceutical and Biological Products (Beijing, China), with a purity＞99.0%. Vancomycin, neomycin sulfate, metronidazole, and ampicillin were obtained from Sangon Biotech (Shanghai, China).

### Animals and Treatments

The six- to eight-week-old BABL/c mice purchased from the Laboratory Animal Center of Southern Medical University were accommodated in specific-pathogen-free conditions for a week with accessible water and food. The animal procedure was carried out according to the National Institutes of Health Guide for the Care and Use of Laboratory Animals of Southern Medical University (Ethical Committee Approval Code: L2018123).

Mice were randomly divided into four groups with eight per group: control, antibiotic, METH, and antibiotic pretreatment. Briefly, based on the METH model of escalating dose/multiple binge, the doses were as follows (Chen et al., 2021): days 1–2, 1.5 mg/kg; days 3–4, 4.5 mg/kg; days 5–6, 7.5 mg/kg; and days 7–8, 10 mg/kg, four injections a day every 2 h. Mice received antibiotics (vancomycin, 100 mg/kg; neomycin sulfate, 200 mg/kg; metronidazole, 200 mg kg; and ampicillin, 200 mg/kg) and were gavaged once a day for 1 week (Gregory et al., 2015; Hu et al., 2017), and the following week, they were simultaneously injected with METH as mentioned above. All mice were deeply anesthetized with pentobarbital (45 mg/kg i.p.) within 24 h of the final dose. Blood samples were collected and centrifuged (4°C, 1,2000 r/min, 10 min) and then stored at −80°C until biochemical analysis. The liver tissues were removed and weighed, a portion was fixed with formalin buffered with 10% PBS, and others were stored at −80°C for further analysis.

### Histological Analysis: Determination of Aspartate Transaminase and Alanine Aminotransferase Levels in Serum

The liver tissue was dehydrated, embedded in paraffin, sectioned (3 μm thickness), and stained with hematoxylin and eosin (H&E) (Xie et al., 2019). The pathological morphology of liver tissue was observed under an optical microscope (Olympus, Tokyo, Japan). The activities of serum alanine aminotransferase (ALT) and aspartate aminotransferase (AST) were measured with the ELISA kit (MEIMIAN, Jiangsu, China) following the manufacturer’s instructions.

### Biochemical Analysis in Liver

Liver tissue (not less than 100 mg) was homogenized with 200 μl PBS (pH = 7.2–7.4, concentration 0.01 mol/L) and centrifuged (12,000 g/min) at 4°C for 10 min. The supernatant was taken to measure protein concentration using the BCA kit (Thermo Scientific, MA, United States). SOD and ROS concentrations in liver tissue were detected using the ELISA kit (MEIMIAN, Jiangsu, China) following the reagent protocol.

### RNA-seq Analyses

#### RNA Extraction, Library Preparation, and Illumina HiSeq X Ten/NovaSeq 6000 Sequencing

Total RNA of liver was extracted with TRIzol® reagent (Invitrogen), and gDNA was removed using DNase I (TaKara). The RNA-seq transcriptome library was prepared using 1 μg total RNA according to TruSeq™ RNA Sample Preparation Kit from Illumina (San Diego, CA, United States). The RNA-seq process is as follows: isolate mRNA, fragment mRNA, synthesize cDNA, connect adaptor, select fragment, PCR cycles, and Illumina HiSeq X Ten/NovaSeq 6000 sequence.

### Read Mapping

Quality control of raw data was conducted with SeqPrep (https://github.com/jstjohn/SeqPrep) and Sickle (https://github.com/najoshi/sickle). The clean reads were separately compared with the reference genome using HISAT2 software (http://ccb.jhu.edu/software/hisat2/index.shtml) (Kim et al., 2015). StringTie (https://ccb.jhu.edu/software/stringtie/index.shtml? t=example) was used to assemble the mapped reads of each sample in a reference-based approach (Pertea et al., 2015).

### Differential Expression Analysis and Functional Enrichment

To identify the difference of DEGs among different samples, the transcripts per million reads (TPM) method calculated the expression level of each transcript.

DEGs with fold change ≥2 or ≤0.5 were considered to be significantly differentially expressed genes. GO functional enrichment and KEGG pathway analysis were carried out through Goatools (https://github.com/tanghaibao/Goatools) and KOBAS (http://kobas.cbi.pku.edu.cn/home.do) (Xie et al., 2011). PPI analysis was conducted with Cytoscape 3.7.1 software.

### Real-Time Quantitative Polymerase Chain Reaction Analysis

To verify RNA-seq results, four genes (Acaca, Chrna4, Nr1d2, and Csrnp1) were randomly selected for real-time quantitative PCR (RT-qPCR) analysis. Total RNA was extracted with TRIzol® reagent (Invitrogen, MA, United States), and RNA quality was detected with a NanoDrop 2000 Spectrophotometer (Thermo Scientific, MA, United States) (Zhou et al., 2020). Total RNA was converted to cDNA using Hifair™ II First-Strand cDNA Synthesis SuperMix (YEASEN, Shanghai, China). Real-time quantitative PCR (RT-qPCR) was performed on a LightCycler® 96 System (Roche Life Science, Penzberg, Germany) using Hieff™ qPCR SYBR Green Master Mix (No Rox Plus, YEASEN, Shanghai, China) to quantify mRNA expression. Primers were designed with Primer3 web software (http://primer3.ut.ee/) (Table 1). The gene expression was normalized to β-actin. Relative quantification was calculated using the CT (2^–ΔΔCt^) method.

**TABLE 1:** Primers for RT-qPCR analysis.

Gene	F	R
Csrnp1	5′-TTC​ATT​CAC​ACC​CTC​ACC​CG-3′	5′-CCA​GGG​AGG​CTA​CCT​TCT​CT-3′
Nr1d2	5′-CCA​CCT​GCA​GAA​TGA​CAC​C-3′	5′-AGA​TGC​ATC​CTC​CCT​CCA​GT-3′
Chrna4	5′-CTG​CCC​CAA​CTT​TCT​GCA​AC-3′	5′-TGG​CCA​CGT​ATT​TCC​AGT​CC-3′
Acaca	5′-TTG​CCA​TGG​GGA​TCC​CTC​TA-3′	5′-GCT​GTT​CCT​CAG​GCT​CAC​AT-3′
β-Actin	5′-CTG​GTC​GTA​CCA​CAG​GCA​TT-3′	5′-TGC​TAG​GAG​CCA​GAG​CAG​TA-3′

### Western Blot Analysis

The total liver tissue protein was extracted with lysis buffer (RIPA lysis buffer: PMSF: phosphatase inhibitors = 100: 1: 1). The Pierce™ BCA Protein Assay Kit (Thermo Scientific, MA, United States) was used to measure the protein concentration. 20 μg of proteins was resolved by 12% SDS-PAGE and transferred to PVDF membranes. The membranes were blocked with 5% skim milk at room temperature for 2 h, incubated with primary antibodies overnight at 4°C, and then incubated with appropriate secondary antibodies for 2 h at room temperature. Protein bands were revealed using chemiluminescence reagents (Thermo Scientific, MA, United States). The grayscale was calculated by ImageJ (version 1.8.0) software with normalization to that of β-actin. The antibodies and their dilutions were as follows: TLR4 (1:1,000; Santa Cruz, CA, United States), MyD88 (1:1,000; CST), Traf6 (1:1,000; Santa Cruz, CA, United States), and GAPDH (1:1,000; Proteintech).

### Statistical Analysis

All results were reported as mean ± SEM. Statistical analysis was performed using GraphPad Prism 8.0 software. Statistical significance was calculated using one-way ANOVA followed by the Bonferroni post hoc test for multiple comparisons. *p*-Values < 0.05 were considered statistically significant (*/#*p* < 0.05, **/##*p* < 0.01, and ***/###*p* < 0.001).

## Results

### Antibiotics Attenuated Methamphetamine-Induced Hepatotoxicity

**FIGURE 1:**
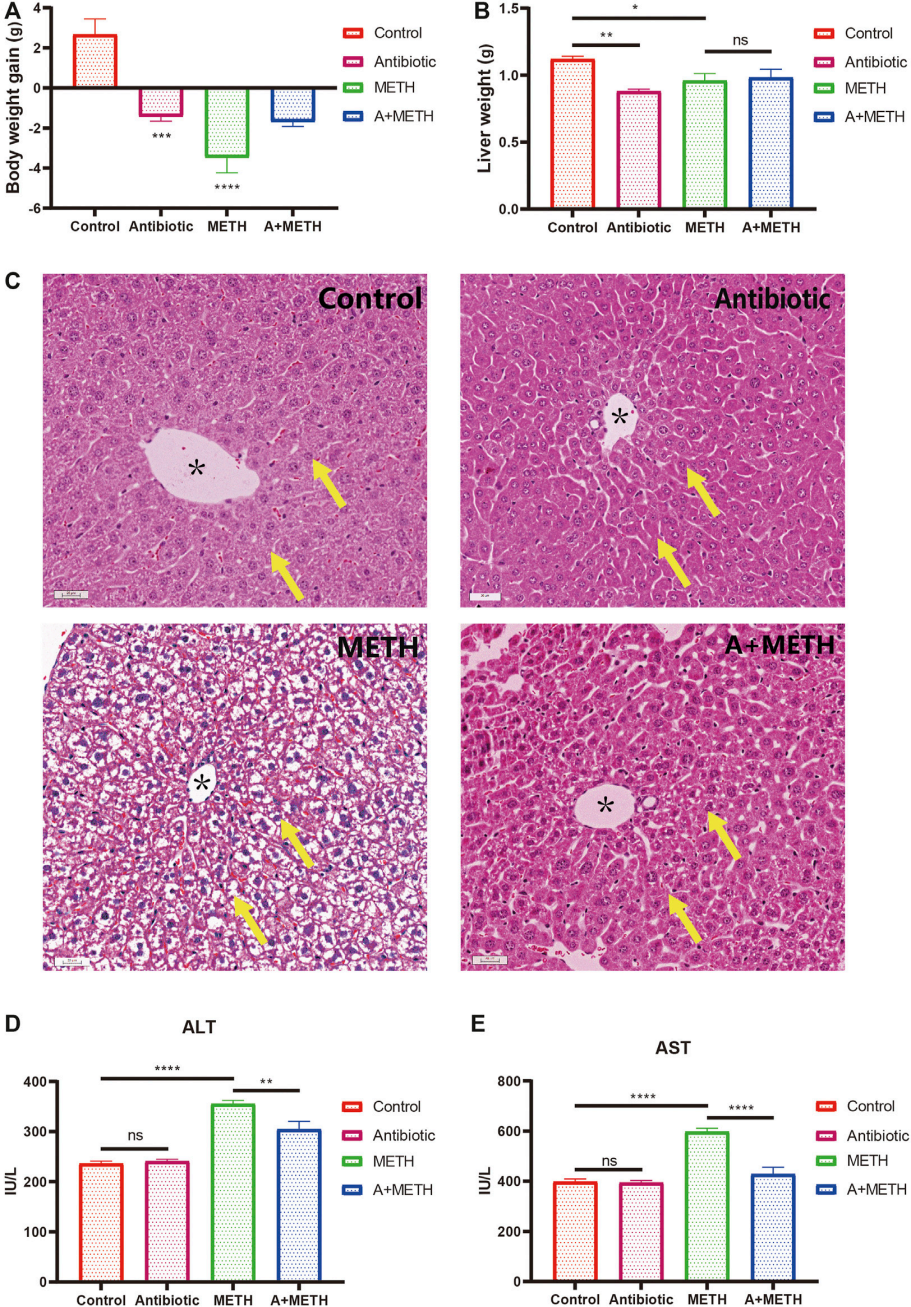
Changes in body weight **(A)** and liver weight **(B)** of mice compared with the control group. Histopathological assessment of METH liver injury **(C)**. The yellow arrows point to the nucleus and cytoplasm; * indicates the central vein; bar = 20 μm, 20×. METH significantly enhanced serum ALT **(D)** and AST **(E)** activities relative to the control group. The increasing level was attenuated in the A + METH (antibiotic + METH) group. *p < 0.05; **p < 0.01; ***p < 0.001; ns, statistically non-significant.

Antibiotic and METH treatment significantly decreased the body and liver weights of mice (Figures 1A,B). Antibiotic pretreatment tends to alleviate METH‐induced body and liver weight loss, with a non-significant difference (Figures 1A,B). Histopathological analysis revealed that karyopyknosis and extensive cytoplasmic damage (vacuolar degeneration) in METH-treated mice were ameliorated by antibiotic pretreatment (Figure 1C). However, there is no noticeable damage in hepatocytes’ morphology in control and antibiotic groups (Figure 1C). Similarly, serum ALT and AST activities were remarkably increased after METH administration but decreased with antibiotic pretreatment (Figures 1D,E).

### Antibiotic Pretreatment Mitigated Methamphetamine-Induced ROS and SOD Levels

**FIGURE 2:**
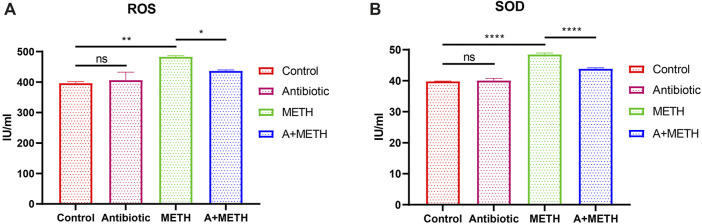
Analyses of ROS and SOD activities. The treatment with METH significantly increased ROS **(A)** and SOD **(B)** activities, which significantly repressed with antibiotic pretreatment. Antibiotics alone did not change compared to the control group. *p < 0.05; **p < 0.01; ***p < 0.001; ns, statistically non-significant.

To further investigate the protective effect of antibiotics on METH-induced liver injury, ROS and SOD concentrations in liver were measured. As illustrated in Figure 2, antibiotic cocktail pretreatment dramatically relieved METH-induced ROS and SOD elevation activities.

### RNA-seq

#### Principal Correlation Analysis and Differentially Expressed Genes

**FIGURE 3:**
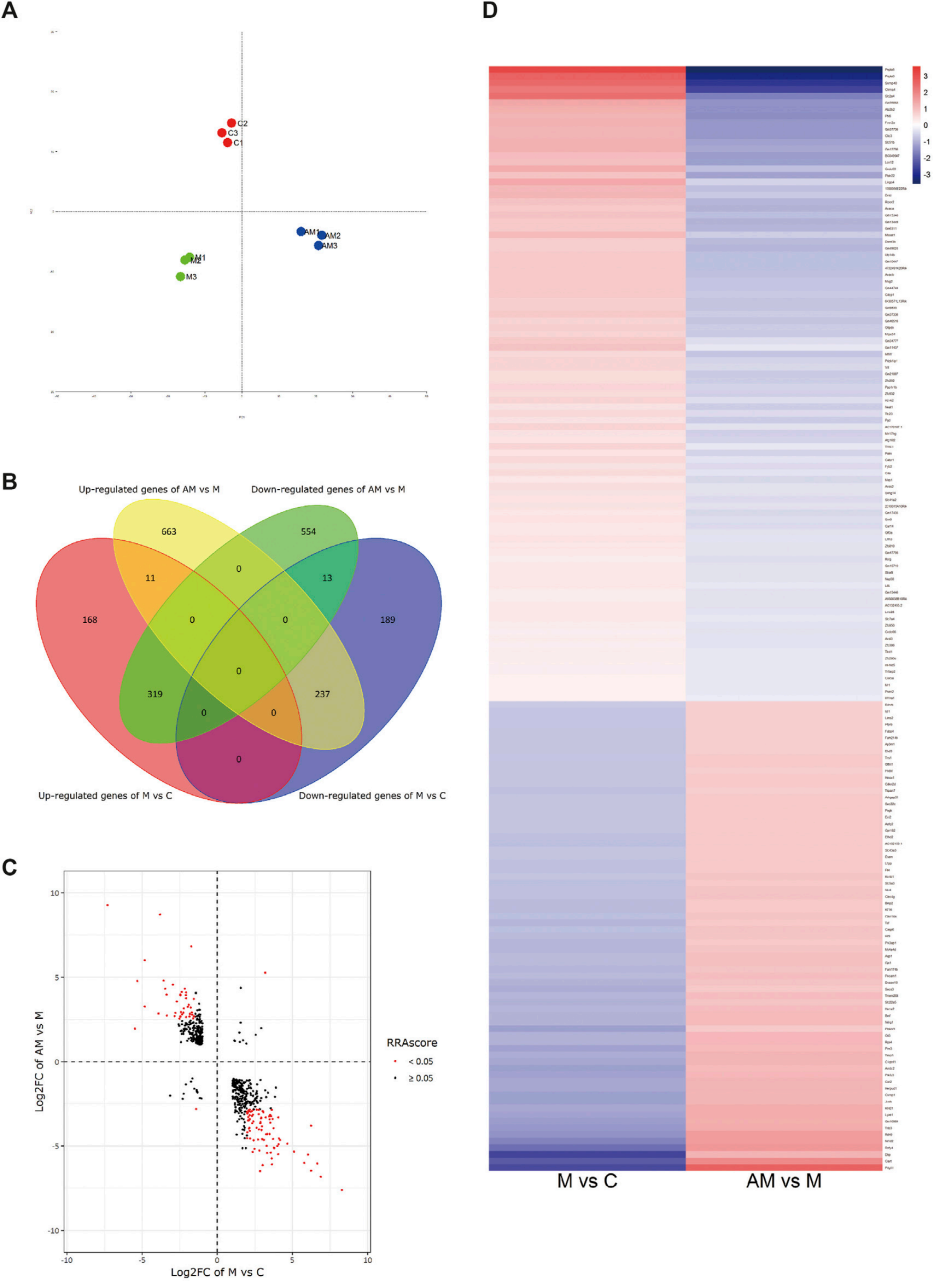
**(A)** Principal correlation analysis. **(B)** Venn diagram of DEGs. **(C)** Volcano plot diagram: the first and third quadrants show the genes both up- and downregulated in both METH and antibiotic-pretreated groups, the second quadrant shows the genes downregulated in the METH group but upregulated in the AM group, and the fourth quadrant shows the genes upregulated in the METH group but downregulated in the AM group. **(D)** Heatmap diagram of DEG analysis among groups. The blue color represents downregulation, while red represents upregulation. C, control group; M, METH group; AM, antibiotic + METH group.

PCA intuitively showed a clear correlation between the gene expression of samples in the group and apparent separation and distinction among the three groups (Figure 3A). A total of 580 DEGs were identified (Ⅰlog_2_ (fold change)Ⅰ≥ 1), of which 319 were upregulated after METH treatment and then downregulated with antibiotic pretreatment and 237 were first downregulated after METH administration and then upregulated after antibiotic pretreatment, in addition to 11 upregulated and 13 downregulated ones simultaneously in METH and antibiotic-pretreated groups (Figures 3B–D).

#### GO and KEGG Pathway Enrichment Analyses

**FIGURE 4:**
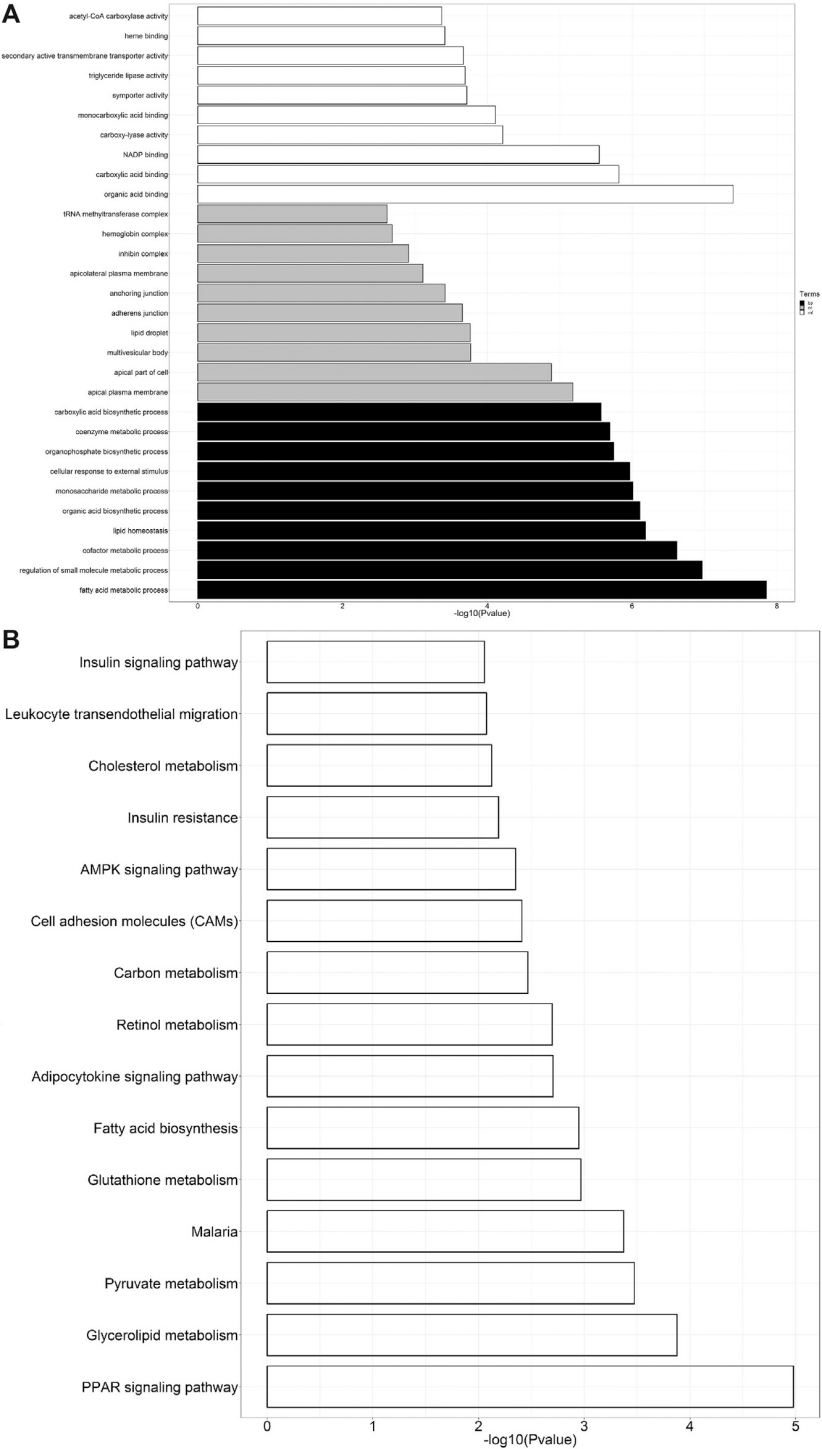
**(A)** GO analysis of DEGs. The black bars represent biological processes (bp), gray represents cellular components (cc), and white represents molecular functions (mf). **(B)** KEGG pathway enrichment analysis of DEGs.

To further predict the relationship between biological function and DEGs, GO analysis was carried out in three categories: molecular function, biological process, and cell composition (Figure 4A). KEGG analysis revealed that 580 DEGs were enriched in 15 pathways (Figure 4B), contributing to the study of gene and expression information of the whole network. Figure 4B displays enrichment of KEGG pathways, including AMPK and PPAR signaling pathways, fatty acid biosynthesis, and cholesterol and retinol metabolisms.

#### PPI Analysis

**FIGURE 5:**
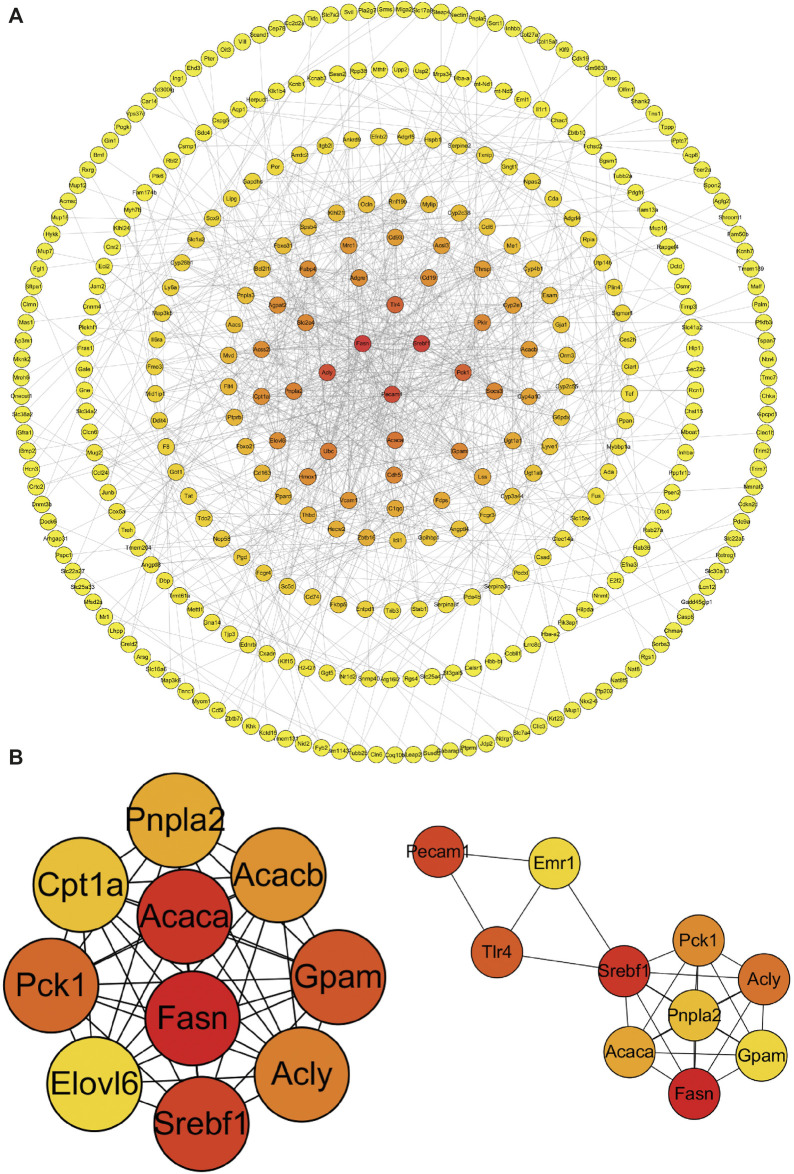
PPI network **(A)** and hub genes **(B)** of all DEGs identified among the three groups. Red balls show the hub genes. PPI, protein–protein interaction.

**FIGURE 6:**
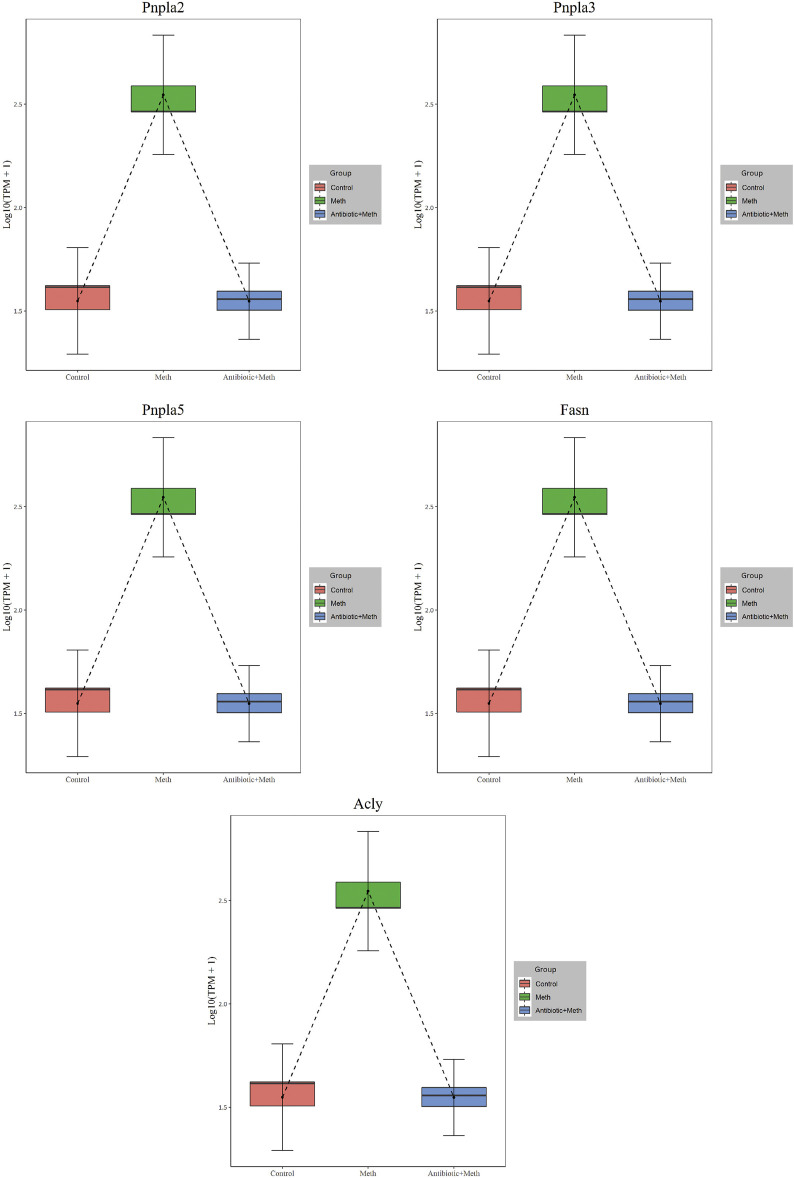
Expression of hub genes in RNA-seq.

The PPI analysis allows us to better understand molecular mechanisms of core signaling pathways and core genes (Fei et al., 2020). The more connections or the closer to circle center of genes might be related to underlying mechanisms of METH hepatotoxicity and antibiotic protection (Figure 5A). The hub genes corresponding to the top-ranked proteins were selected by Cytoscape software, and these genes mostly interacted with other DEGs such as Pnpla2, Pnpla5, Pnpla3, Fasn, Acly, and TLR4 (Figure 5B, Figure 6).

### Validation of the RNA-seq Results via Real-Time Quantitative Polymerase Chain Reaction

**FIGURE 7:**
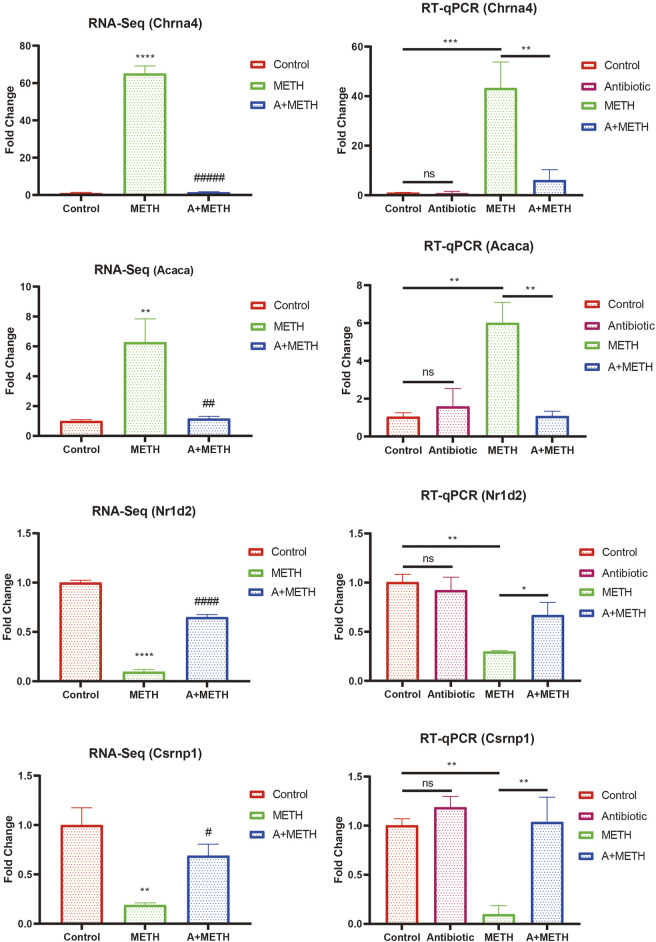
Consistency of RNA-seq and RT-qPCR results. RT-qPCR results of selected DEGs (Chrna4, Acaca, Nr1d2, and Csrnp1) were consistent with those of RNA-seq. RNA-seq: *p < 0.05, **p < 0.01, ***p < 0.001, significantly different compared to the control group; #*p* < 0.05, ##*p* < 0.01, ###*p* < 0.001, significantly different compared to the METH group. RT-qPCR: *p < 0.05, **p < 0.01, ***p < 0.001, ns, statistically non-significant.

To verify RNA-seq results’ accuracy, four genes (Chrna4, Acaca, Nr1d2, and Csrnp1) were randomly selected for RT-qPCR analysis. Acaca and Chrna4 were upregulated DEGs in the METH group compared with the control group and downregulated after antibiotic pretreatment, while Nr1d2 and Csrnp1 were reversed. The RT-qPCR results followed those of RNA-seq (Figure 7). However, RNA-seq was not conducted in the antibiotic group, and RT-qPCR data certified no difference compared to the control group.

### Pretreatment of Antibiotics Alleviates Methamphetamine-Induced Inflammation via TLR4/MyD88/Traf6 Pathway

**FIGURE 8:**
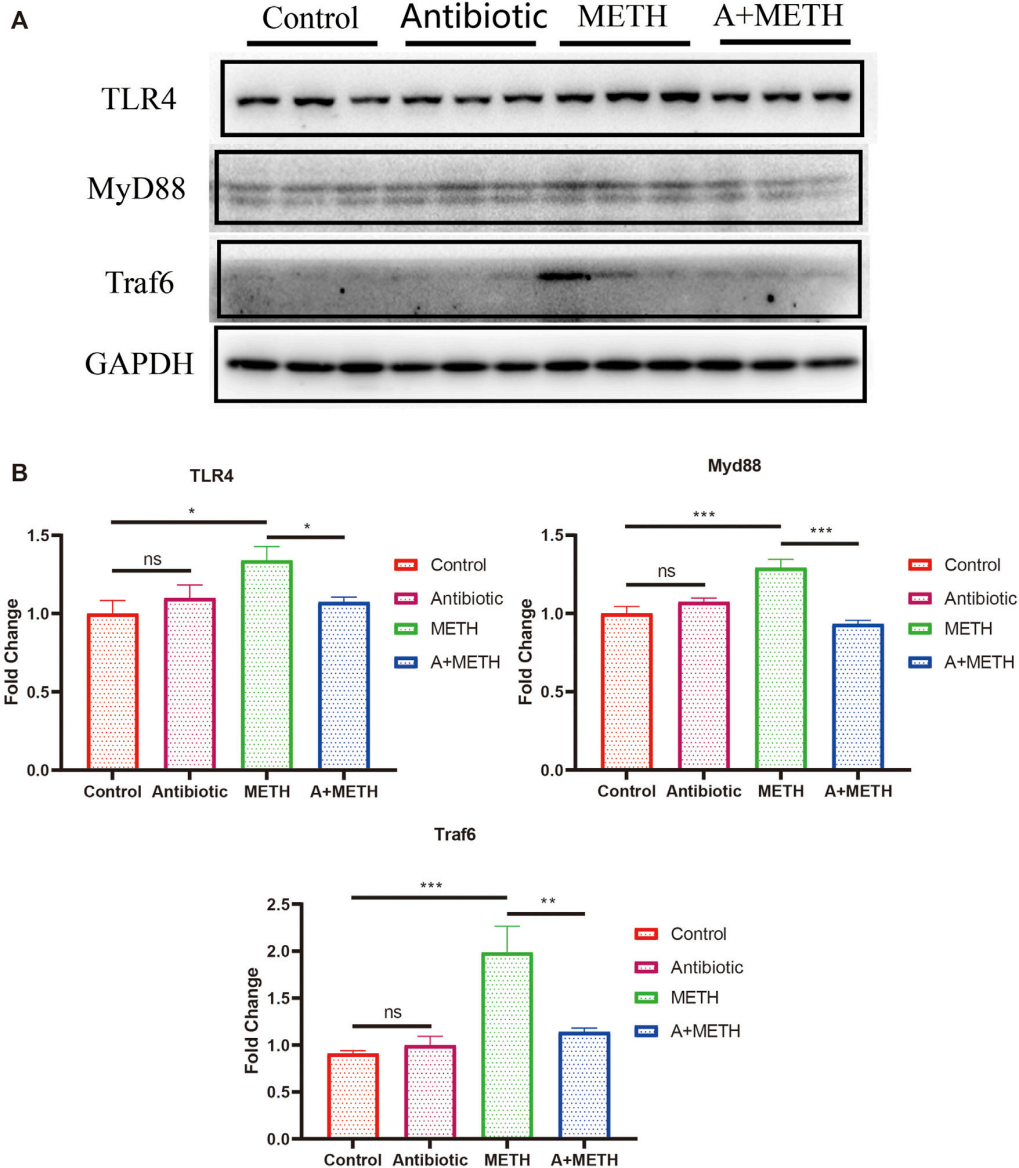
**(A)** Western blots showing the expression of TLR4, MyD88, and Traf6 proteins in the mice liver. **(B)** The bar chart presents the statistical analysis of TLR4, MyD88, and Traf6 protein expressions. *p < 0.05; **p < 0.01; ***p < 0.001; ns, statistically non-significant.

As indicated by PPI analysis, TLR4 is one of the hub genes, which may have a strong association with METH-induced hepatotoxicity. As shown in Figure 8, protein levels of some key factors in the TLR4/MyD88-dependent signaling axis, including TLR4, MyD88, and Traf6, were upregulated in the METH-treated group, and antibiotic pretreatment effectively inhibited upregulation, while control and antibiotic groups showed no difference.

## Discussion

It has been confirmed that METH induces hepatotoxicity in humans, rats, and cells (Dias Da Silva et al., 2013; Zhao et al., 2020; Zhang et al., 2021). Consistent with findings, this research demonstrated that the escalating dose/multiple binge METH model could also lead to hepatotoxicity in mice, indicated by histopathological examination and ALT and AST activities. The histopathology visually showed METH-induced hepatotoxicity, including nuclear pyknosis and cytoplasmic vacuolation. ALT and AST activity levels in serum are biomarkers for evaluating liver function. After METH injection, these markers increased, suggesting that METH resulted in liver dysfunction. Collectively, antibiotic pretreatment improved abnormalities alteration above, demonstrating that antibiotics can protect the liver from METH damage.

Oxidative stress is characterized by imbalanced generation of reactive oxygen species (ROS) and activity of antioxidant defenses (Bresciani et al., 2015; Arefin et al., 2020) and is known to be related to pathologies of some diseases, such as atherosclerosis (Kattoor et al., 2017), Alzheimer’s disease (Chen and Zhong, 2014), and tumorigenesis (Gorrini et al., 2013). Oxidative stress is now recognized as a contributing factor in pathogenesis of various liver diseases. Alcoholic liver disease (ALD), non-alcoholic fatty liver disease (NAFLD), hepatic encephalopathy (HE), hepatic fibrosis, and hepatitis C virus (HCV) are undoubtedly linked to ROS overproduction and existence of oxidative stress within hepatocytes (Cichoż-Lach, 2014). Superoxide dismutase (SOD) is an important superoxide and free radical scavenger that controls the levels of all kinds of ROS and reactive nitrogen species (Halliwell and Gutteridge, 1984; Wang et al., 2018). SOD levels in serum and liver tissues are significantly increased in acute liver failure patients than in healthy controls, which may be an adaptive response to limit ROS harmful effects (Tian et al., 2018). Consistent with previous studies (Xie et al., 2018a; Zhang et al., 2018), this study reported that ROS and SOD levels were increased after METH treatment. Poorly absorbed antibiotics in drinking water were reported to reverse age‐related arterial dysfunction, inflammation, and oxidative stress in mice by inhibiting intestinal flora (Brunt et al., 2019). The generation of ROS may be related to the gut microbiota (Jones and Neish, 2017), and gut microbiota–derived short-chain fatty acids (SCFAs) affect mitochondrial function and reduce ROS production (Mottawea et al., 2016; Luca et al., 2019). The present study also proved that antibiotic pretreatment could prevent METH-induced oxidative stress by inhibiting the increase of ROS and SOD, which may be the potential mechanism.

RNA-seq was also used to further investigate the underlying mechanism of protective effect of antibiotics against METH-induced hepatotoxicity. Hundreds of DEGs have been detected by sequencing, and we screened out some of the hub genes for subsequent verification; meanwhile, subsequent GO and KEGG analyses were performed based on these DEGs. The AMPK signaling pathway was reported to reverse steatosis and inflammation in non-alcoholic fatty liver disease (NAFLD) (Li et al., 2019). The PPAR signaling pathway plays an important role in attenuating liver fibrosis (Panebianco et al., 2017) and hepatic lipoinflammation (Ishtiaq et al., 2019). This research and our previous studies about METH-induced hepatotoxicity in rats (Koriem and Soliman, 2014; Wang et al., 2017) indicated that AMPK and PPAR signaling pathways were enriched in KEGG analysis, highlighting their possible significant correlation with METH-induced hepatotoxicity, though further research is required.

Toll-like receptors (TLRs) hold a fundamental function in regulating innate and adaptive immune response, pathogen recognition, and inflammatory responses (Lai et al., 2015; Xie et al., 2018b). TLR4 can recruit the adaptor proteins (such as MyD88), bind to Traf6, and eventually result in inflammation (Xie et al., 2018b). Interestingly, RNA-seq data suggested that TLR4 is one of the hub genes. The western blot results showed that TLR4/MyD88/Traf6 was elevated after METH treatment, suggesting that METH treatment induced liver inflammation, and antibiotics pretreatment can reverse this elevation. Moreover, excessive ROS production caused tissue damage and accelerated release and development of inflammation via activating the TLR4/MyD88/Traf6 pathway (Gong et al., 2019; Zhou et al., 2020). The above results indicated that antibiotic preconditioning exerts protection against METH-induced hepatotoxicity by inhibiting the TLR4/MyD88/Traf6 pathway.

There is growing evidence showing that alterations in the gut microbiome are linked to pathogenesis and advancement of liver diseases named the “gut–liver axis” (Ma et al., 2017; Milosevic et al., 2019; Albillos et al., 2020; Jones and Neish, 2021). A recent report revealed that intestinal dysbacteriosis might directly cause liver toxicity through the portal vein or destruction of the intestinal barrier, leading to increased bacterial translocation and inflammation (Acharya and Bajaj, 2021). Recent reports have shown that antibiotics positively affect liver injury caused by intestinal bacteria overgrowth, confirming the relationship between intestinal microbiota and liver diseases (Sajjad et al., 2005; Wu et al., 2008). Our previous studies have shown that, after METH treatment, intestinal probiotics were decreased and opportunistic pathogens were increased (Chen et al., 2021). Notably, antibiotics used in this study have been proven to affect clearing intestinal flora (Rakoff-Nahoum et al., 2004; Carvalho et al., 2012). Overall, our analysis reveals a novel perspective that METH can induce hepatotoxicity in mice, and the gut–liver axis may mediate the protective impact of antibiotic pretreatment.

Some limitations are found in this study. First, RNA-seq was not performed in the antibiotic group based on histopathology results, serological indexes, and oxidative stress. Antibiotics alone cannot cause liver damage. Subsequently, we verified RNA-seq results by RT-qPCR, and the gene changes after antibiotic treatment were consistent with those in the control group. Second, intestinal flora data lacked in this study, but antibiotics’ effect on intestinal flora clearance has been confirmed. Finally, it has not been verified whether a correlation is found between METH-induced liver damage and intestinal flora. In combination with our previous study and the present study, further research is required to verify the interaction mechanism among intestinal microbiota, nervous system, and liver.

**FIGURE 9:**
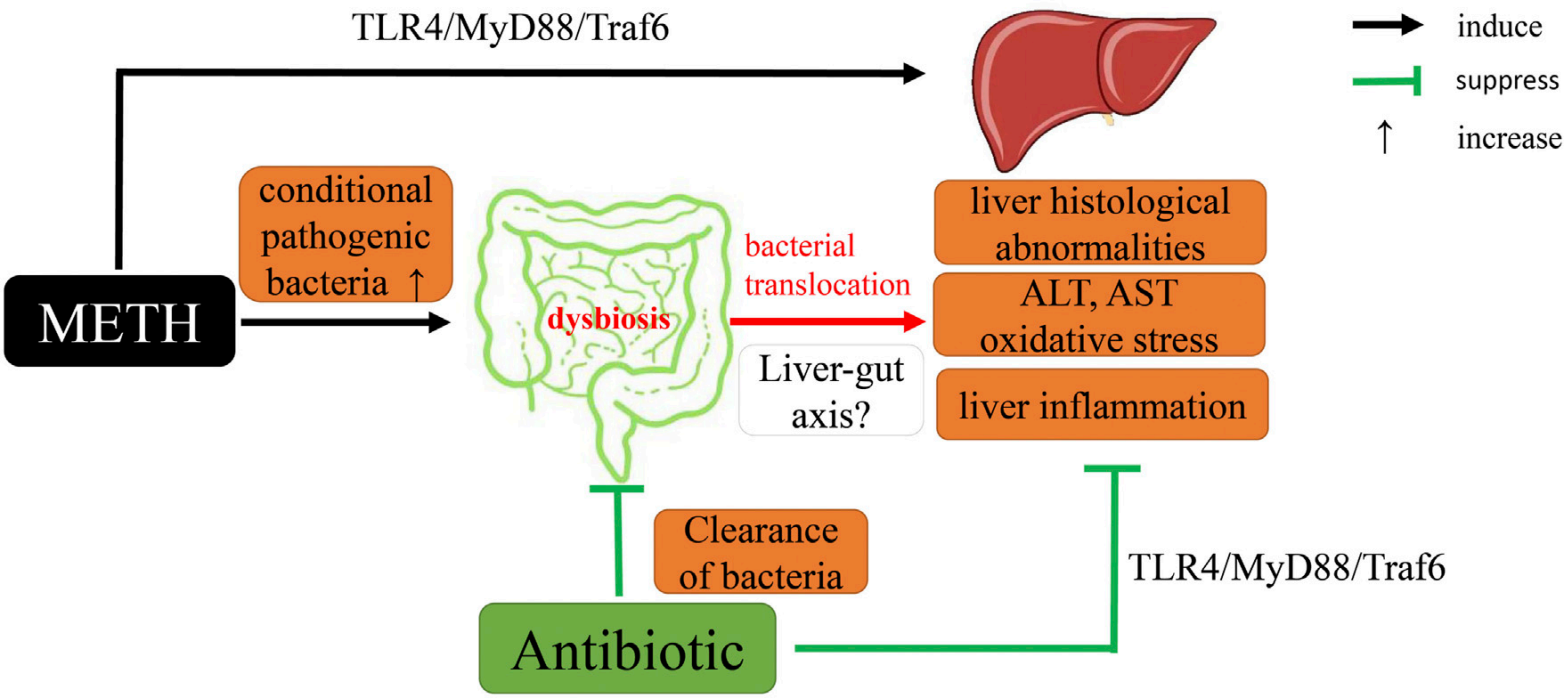
A proposed mechanistic diagram of antibiotic protective action on METH-induced liver hepatotoxicity in this study.

In conclusion, we demonstrated that METH exposure could lead to abnormal pathological and serological changes and activation of oxidative stress and TLR4/MyD88/Traf6 pathway. Gut microbiome clearance by antibiotics alleviated above METH-induced changes. The RNA-seq results provide a possible clue for the basic pathway and antibiotic mechanisms in response to METH. Notably, we first proposed the relationship between METH-induced hepatotoxicity and gut microbiota (Figure 9). Therefore, intestinal microbiota may be a potential therapy for METH-induced hepatotoxicity, though further research is needed to unveil the detailed mechanism.

## Data Availability

The datasets presented in this study can be found in online repositories. The names of the repository/repositories and accession number(s) can be found below: NCBI SRA BioProject, accession no. PRJNA741711.

## References

[B1] Acharya C. Bajaj J. S. (2021). Chronic Liver Diseases and the Microbiome-Translating Our Knowledge of Gut Microbiota to Management of Chronic Liver Disease. Gastroenterology 160 (2021), 556–572. 10.1053/j.gastro.2020.10.056 33253686 PMC9026577

[B2] Albhaisi S. A. M. Bajaj J. S. Sanyal A. J. (2020). Role of Gut Microbiota in Liver Disease. Am. J. Physiol. Gastrointest. Liver Physiol. 318, G84–G98. 10.1152/ajpgi.00118.2019 31657225

[B3] Albillos A. de Gottardi A. Rescigno M. (2020). The Gut-Liver axis in Liver Disease: Pathophysiological Basis for Therapy. J. Hepatol. 72, 558–577. 10.1016/j.jhep.2019.10.003 31622696

[B4] Albillos A. (2003). Increased Lipopolysaccharide Binding Protein in Cirrhotic Patients with Marked Immune and Hemodynamic Derangement. Hepatology 37, 208–217. 10.1053/jhep.2003.50038 12500206

[B5] Arefin S. Buchanan S. Hobson S. Steinmetz J. Alsalhi S. Shiels P. G. (2020). Nrf2 in Early Vascular Ageing: Calcification, Senescence and Therapy. Clin. Chim. Acta 505, 108–118. 10.1016/j.cca.2020.02.026 32097628

[B6] Bajaj J. S. Barbara G. DuPont H. L. Mearin F. Gasbarrini A. Tack J. (2018). New Concepts on Intestinal Microbiota and the Role of the Non-absorbable Antibiotics with Special Reference to Rifaximin in Digestive Diseases. Dig. Liver Dis. 50, 741–749. 10.1016/j.dld.2018.04.020 29807873

[B7] Berg R. D. Garlington A. W. (1979). Translocation of Certain Indigenous Bacteria from the Gastrointestinal Tract to the Mesenteric Lymph Nodes and Other Organs in a Gnotobiotic Mouse Model. Infect. Immun. 23, 403–411. 10.1128/IAI.23.2.403-411.1979 154474 PMC414179

[B8] Bresciani G. da Cruz I. B. M. González-Gallego J. (2015). Manganese Superoxide Dismutase and Oxidative Stress Modulation. Adv. Clin. Chem., 68:87–130. 10.1016/bs.acc.2014.11.001 25858870

[B9] Brunt V. E. Gioscia‐Ryan R. A. Richey J. J. Zigler M. C. Cuevas L. M. Gonzalez A. (2019). Suppression of the Gut Microbiome Ameliorates Age‐related Arterial Dysfunction and Oxidative Stress in Mice. J. Physiol. 597, 2361–2378. 10.1113/JP277336 30714619 PMC6487935

[B10] Carvalho B. M. Guadagnini D. Tsukumo D. M. L. Schenka A. A. Latuf-Filho P. Vassallo J. (2012). Modulation of Gut Microbiota by Antibiotics Improves Insulin Signalling in High-Fat Fed Mice. Diabetologia 55, 2823–2834. 10.1007/s00125-012-2648-4 22828956

[B11] Chen L.-J. Zhi X. Zhang K.-K. Wang L.-B. Li J.-H. Liu J.-L. (2021). Escalating Dose-Multiple Binge Methamphetamine Treatment Elicits Neurotoxicity, Altering Gut Microbiota and Fecal Metabolites in Mice. Food Chem. Toxicol. 148, 111946. 10.1016/j.fct.2020.111946 33359793

[B12] Chen Z. Zhong C. (2014). Oxidative Stress in Alzheimer's Disease. Neurosci. Bull. 30, 271–281. 10.1007/s12264-013-1423-y 24664866 PMC5562667

[B13] Cichoż-Lach H. (2014). Oxidative Stress as a Crucial Factor in Liver Diseases. Wjg 20, 8082. 10.3748/wjg.v20.i25.8082 25009380 PMC4081679

[B14] Dias Da Silva D. Carmo H. Lynch A. Silva E. (2013). An Insight into the Hepatocellular Death Induced by Amphetamines, Individually and in Combination: the Involvement of Necrosis and Apoptosis. Arch. Toxicol. 87, 2165–2185. 10.1007/s00204-013-1082-9 23820845

[B15] Eskandari M. R. Rahmati M. Khajeamiri A. R. Kobarfard F. Noubarani M. Heidari H. (2014). A New Approach on Methamphetamine-Induced Hepatotoxicity: Involvement of Mitochondrial Dysfunction. Xenobiotica 44, 70–76. 10.3109/00498254.2013.807958 23786375

[B16] Fei H. Chen S. Xu C. (2020). RNA-sequencing and Microarray Data Mining Revealing: the Aberrantly Expressed mRNAs Were Related with a Poor Outcome in the Triple Negative Breast Cancer Patients. Ann. Transl Med. 8, 363. 10.21037/atm.2020.02.51 32355807 PMC7186670

[B17] Gong T. Jiang W. Gao Z. Chen Y. Gao S. (2019). Dibromoacetic Acid Induced Hepatotoxicity in Mice through Oxidative Stress and Toll-like Receptor 4 Signaling Pathway Activation. Oxid. Med. Cell Longevity 2019, 1–10. 10.1155/2019/5637235 PMC6886355 31827682

[B18] Gorrini C. Harris I. S. Mak T. W. (2013). Modulation of Oxidative Stress as an Anticancer Strategy. Nat. Rev. Drug Discov. 12, 931–947. 10.1038/nrd4002 24287781

[B19] Gregory J. C. Buffa J. A. Org E. Wang Z. Levison B. S. Zhu W. (2015). Transmission of Atherosclerosis Susceptibility with Gut Microbial Transplantation. J. Biol. Chem. 290, 5647–5660. 10.1074/jbc.M114.618249 25550161 PMC4342477

[B20] Halliwell B. Gutteridge J. M. C. (1984). Oxygen Toxicity, Oxygen Radicals, Transition Metals and Disease. Biochem. J. 219, 1–14. 10.1042/bj2190001 6326753 PMC1153442

[B21] Hashimoto K. Ishima T. Fujita Y. Zhang L. (2013). [Antibiotic Drug Minocycline: a Potential Therapeutic Drug for Methamphetamine-Related Disorders], Nihon Arukoru Yakubutsu Igakkai Zasshi, 48, 118–125. 23808319

[B22] Hashimoto K. (2008). Minocycline as a Therapeutic Drug for Methamphetamine Use Disorders. Nihon Shinkei Seishin Yakurigaku Zasshi, 28, 19–22. 18411705

[B23] Hu J. Luo H. Wang J. Tang W. Lu J. Wu S. (2017).Enteric Dysbiosis-Linked Gut Barrier Disruption Triggers Early Renal Injury Induced by Chronic High Salt Feeding in Mice. Exp. Mol. Med., 49, e370. 10.1038/emm.2017.122 28857085 PMC5579512

[B24] Ishtiaq S. M. Rashid H. Hussain Z. Arshad M. I. (2019). Adiponectin and PPAR: a Setup for Intricate Crosstalk between Obesity and Non-alcoholic Fatty Liver Disease. Rev. Endocr. Metab. Dis., 253–261. 10.1007/s11154-019-09510-2 31656991

[B25] Jones R. M. Neish A. S. (2021). Gut Microbiota in Intestinal and Liver Disease. Annu. Rev. Pathol. Mech. Dis. 16, 251–275. 10.1146/annurev-pathol-030320-095722 33234022

[B26] Jones R. M. Neish A. S. (2017). Redox Signaling Mediated by the Gut Microbiota. Free Radic. Biol. Med. 105, 41–47. 10.1016/j.freeradbiomed.2016.10.495 27989756

[B27] Kim D. Langmead B. Salzberg S. L. (2015). HISAT: a Fast Spliced Aligner with Low Memory Requirements. Nat. Methods 12, 357–360. 10.1038/nmeth.3317 25751142 PMC4655817

[B28] Koriem K. M. M. Soliman R. E. (2014). Chlorogenic and Caftaric Acids in Liver Toxicity and Oxidative Stress Induced by Methamphetamine. J. Toxicol. 2014, 1–10. 10.1155/2014/583494 PMC4127234 25136360

[B29] Lai L. Chen Y. Tian X. Li X. Zhang X. Lei J. (2015). Artesunate Alleviates Hepatic Fibrosis Induced by Multiple Pathogenic Factors and Inflammation through the Inhibition of LPS/TLR4/NF-κB Signaling Pathway in Rats. Eur. J. Pharmacol. 765, 234–241. 10.1016/j.ejphar.2015.08.040 26318197

[B30] Halpin L. E. Gunning W. T. Yamamoto B. K. (2013). Methamphetamine Causes Acute Hyperthermia‐dependent Liver Damage. Pharmacol. res. perspect. 1:e00008. 10.1002/prp2.8 25505562 PMC4184573

[B31] Li C.-X. Gao J.-G. Wan X.-Y. Chen Y. Xu C.-F. Feng Z.-M. (2019). Allyl Isothiocyanate Ameliorates Lipid Accumulation and Inflammation in Nonalcoholic Fatty Liver Disease via the Sirt1/AMPK and NF-Κb Signaling Pathways. Wjg 25, 5120–5133. 10.3748/wjg.v25.i34.5120 31558861 PMC6747284

[B32] Liu Y. Hao B. Shi Y. Xue L. Wang X. Chen Y. (2017). Violent Offences of Methamphetamine Users and Dilemmas of Forensic Psychiatric Assessment, Forensic Sci. Res., 2. 11–17. 10.1080/20961790.2017.1287155 30483614 PMC6197090

[B33] Luca M. Di Mauro M. Di Mauro M. Luca A. (2019). Gut Microbiota in Alzheimer's Disease, Depression, and Type 2 Diabetes Mellitus: The Role of Oxidative Stress. Oxidative Med. Cell Longev. 2019, 1–10. 10.1155/2019/4730539 PMC6501164 31178961

[B34] Ma J. Zhou Q. Li H. (2017). Gut Microbiota and Nonalcoholic Fatty Liver Disease: Insights on Mechanisms and Therapy. Nutrients 9, 1124. 10.3390/nu9101124 PMC5691740 29035308

[B35] Mendoza Y. P. Rodrigues S. G. Bosch J. Berzigotti A. (2020). Effect of Poorly Absorbable Antibiotics on Hepatic Venous Pressure Gradient in Cirrhosis: A Systematic Review and Meta-Analysis. Dig. Liver Dis. 52, 958–965. 10.1016/j.dld.2020.06.048 32736898

[B36] Meng X. Li S. Li Y. Gan R.-Y. Li H.-B. (2018). Gut Microbiota's Relationship with Liver Disease and Role in Hepatoprotection by Dietary Natural Products and Probiotics. Nutrients 10, 1457. 10.3390/nu10101457 PMC6213031 30297615

[B37] Milosevic I. Vujovic A. Barac A. Djelic M. Korac M. Radovanovic Spurnic A. (2019). Gut-Liver Axis, Gut Microbiota, and its Modulation in the Management of Liver Diseases: A Review of the Literature. Ijms 20, 395. 10.3390/ijms20020395 PMC6358912 30658519

[B38] Moratalla R. Khairnar A. Simola N. Granado N. García-Montes J. R. Porceddu P. F. (2017). Amphetamine-related Drugs Neurotoxicity in Humans and in Experimental Animals: Main Mechanisms. Prog. Neurobiol. 155, 149–170. 10.1016/j.pneurobio.2015.09.011 26455459

[B39] Mottawea W. Chiang C.-K. Mühlbauer M. Starr A. E. Butcher J. Abujamel T. (2016). Altered Intestinal Microbiota-Host Mitochondria Crosstalk in New Onset Crohn's Disease. Nat. Commun. 7:13419. 10.1038/ncomms13419 27876802 PMC5122959

[B40] Mu C. Zhu W. (2019). Antibiotic Effects on Gut Microbiota, Metabolism, and beyond. Appl. Microbiol. Biotechnol. 103, 9277–9285. 10.1007/s00253-019-10165-x 31701196

[B41] Panebianco C. Oben J. A. Vinciguerra M. Pazienza V. (2017). Senescence in Hepatic Stellate Cells as a Mechanism of Liver Fibrosis Reversal: a Putative Synergy between Retinoic Acid and PPAR-Gamma Signalings. Clin. Exp. Med. 17, 269–280. 10.1007/s10238-016-0438-x 27655446

[B42] Pertea M. Pertea G. M. Antonescu C. M. Chang T.-C. Mendell J. T. Salzberg S. L. (2015). StringTie Enables Improved Reconstruction of a Transcriptome from RNA-Seq Reads. Nat. Biotechnol. 33, 290–295. 10.1038/nbt.3122 25690850 PMC4643835

[B43] Kattoor A. J. Pothineni N. V. K. Palagiri D. Mehta J. L. (2017). Oxidative Stress in Atherosclerosis. Curr. Atheroscler. Rep. 19, 42. 10.1007/s11883-017-0678-6 28921056

[B44] Qu D. Zhang K. Chen L. Wang Q. Wang H. (2020). RNA-sequencing Analysis of the Effect of Luteolin on Methamphetamine-Induced Hepatotoxicity in Rats: a Preliminary Study. PeerJ 8, e8529. 10.7717/peerj.8529 32071822 PMC7007981

[B45] Rakoff-Nahoum S. Paglino J. Eslami-Varzaneh F. Edberg S. Medzhitov R. (2004). Recognition of Commensal Microflora by Toll-like Receptors Is Required for Intestinal Homeostasis. Cell 118, 229–241. 10.1016/j.cell.2004.07.002 15260992

[B46] Sajjad A. Mottershead M. Syn W. K. Jones R. Smith S. Nwokolo C. U. (2005). Ciprofloxacin Suppresses Bacterial Overgrowth, Increases Fasting Insulin but Does Not Correct Low Acylated Ghrelin Concentration in Non-alcoholic Steatohepatitis. Aliment. Pharmacol. Ther. 22, 291–299. 10.1111/j.1365-2036.2005.02562.x 16097995

[B47] Tian Z. Chen Y. Yao N. Hu C. Wu Y. Guo D. (2018). Role of Mitophagy Regulation by ROS in Hepatic Stellate Cells during Acute Liver Failure. Am. J. Physiol.-Gastrointest. Liver Physiol. 315, G374–G384. 10.1152/ajpgi.00032.2018 29648877

[B48] Tripathi A. Debelius J. Brenner D. A. Karin M. Loomba R. Schnabl B. (2018). The Gut-Liver axis and the Intersection with the Microbiome. Nat. Rev. Gastroenterol. Hepatol. 15, 397–411. 10.1038/s41575-018-0011-z 29748586 PMC6319369

[B49] Wang Q. Wei L.-W. Xiao H.-Q. Xue Y. Du S.-H. Liu Y.-G. (2017). Methamphetamine Induces Hepatotoxicity via Inhibiting Cell Division, Arresting Cell Cycle and Activating Apoptosis: *In Vivo* and *In Vitro* Studies. Food Chem. Toxicol. 105, 61–72. 10.1016/j.fct.2017.03.030 28341135

[B50] Wang Y. Branicky R. Noë A. Hekimi S. (2018). Superoxide Dismutases: Dual Roles in Controlling ROS Damage and Regulating ROS Signaling. J. Cel Biol. 217, 1915–1928. 10.1083/jcb.201708007 PMC5987716 29669742

[B51] Wu W.-C. Zhao W. Li S. (2008). Small Intestinal Bacteria Overgrowth Decreases Small Intestinal Motility in the NASH Rats, World J. Gastroenterol., 14. 313–317. 10.3748/wjg.14.313 18186574 PMC2675133

[B52] Xie C. Mao X. Huang J. Ding Y. Wu J. Dong S. (2011). KOBAS 2.0: a Web Server for Annotation and Identification of Enriched Pathways and Diseases. Nucleic Acids Res. 39, W316–W322. 10.1093/nar/gkr483 21715386 PMC3125809

[B53] Xie X.-L. He J.-T. Wang Z.-T. Xiao H.-Q. Zhou W.-T. Du S.-H. (2018). Lactulose Attenuates METH-Induced Neurotoxicity by Alleviating the Impaired Autophagy, Stabilizing the Perturbed Antioxidant System and Suppressing Apoptosis in Rat Striatum. Toxicol. Lett. 289, 107–113. 10.1016/j.toxlet.2018.03.015 29550550

[B54] Xie X.-L. Zhou W.-T. Zhang K.-K. Chen L.-J. Wang Q. (2018). METH-induced Neurotoxicity Is Alleviated by Lactulose Pretreatment through Suppressing Oxidative Stress and Neuroinflammation in Rat Striatum. Front. Neurosci. 12:802. 10.3389/fnins.2018.00802 30450033 PMC6224488

[B55] Xie X.-L. Zhou W.-T. Zhang K.-K. Yuan Y. Qiu E.-M. Shen Y.-W. (2019). PCB52 Induces Hepatotoxicity in Male Offspring through Aggravating Loss of Clearance Capacity and Activating the Apoptosis: Sex-Biased Effects on Rats. Chemosphere. 227, 389–400. 10.1016/j.chemosphere.2019.04.077 31003123

[B56] Xu B. Ye Y. Liao L. (2019). Rapid and Simple Analysis of Amphetamine-type Illegal Drugs Using Excitation-Emission Matrix Fluorescence Coupled with Parallel Factor Analysis, Forensic Sci. Res., 4. 179–187. 10.1080/20961790.2017.1349600 31304446 PMC6610521

[B57] Xu F. Liu L. (2019). Simultaneous Determination of Free Methamphetamine, Pethidine, Ketamine and Tramadol in Urine by Dispersive Liquid-Liquid Microextraction Combined with GC-MS. Forensic Sci. Res., 4. 188–194. 10.1080/20961790.2017.1377386 31304447 PMC6610497

[B58] Yang X. Wang Y. Li Q. Zhong Y. Chen L. Du Y. (2018). The Main Molecular Mechanisms Underlying Methamphetamine- Induced Neurotoxicity and Implications for Pharmacological Treatment. Front. Mol. Neurosci. 11, 186. 10.3389/fnmol.2018.00186 29915529 PMC5994595

[B59] Zhang K.-K. Wang H. Qu D. Chen L.-J. Wang L.-B. Li J.-H. (2021). Luteolin Alleviates Methamphetamine-Induced Hepatotoxicity by Suppressing the P53 Pathway-Mediated Apoptosis, Autophagy, and Inflammation in Rats. Front. Pharmacol. 12:641917. 10.3389/fphar.2021.641917 33679421 PMC7933587

[B60] Zhang K. Zhang Q. Jiang H. Du J. Zhou C. Yu S. (2018). Impact of Aerobic Exercise on Cognitive Impairment and Oxidative Stress Markers in Methamphetamine-dependent Patients. Psychiatry Res. 266, 328–333. 10.1016/j.psychres.2018.03.032 29588062

[B61] Zhang Z. Gong Q. Feng X. Zhang D. Quan L. (2017). Astrocytic Clasmatodendrosis in the Cerebral Cortex of Methamphetamine Abusers, Forensic Sci. Res., 2, 139–144. 10.1080/20961790.2017.1280890 30483632 PMC6197099

[B62] Zhao T. Zhai C. Song H. Wu Y. Ge C. Zhang Y. (2020). Methamphetamine-Induced Cognitive Deficits and Psychiatric Symptoms Are Associated with Serum Markers of Liver Damage. Neurotox. Res. 37, 67–76. 10.1007/s12640-019-00115-w 31691188

[B63] Zhou W.-T. Wang L.-B. Yu H. Zhang K.-K. Chen L.-J. Wang Q. (2020). N-acetylcysteine Alleviates PCB52-Induced Hepatotoxicity by Repressing Oxidative Stress and Inflammatory Responses. PeerJ (San Francisco, CA) 8, e9720. 10.7717/peerj.9720 PMC7427542 32864221

